# Rates and Predictors of Non-Adherence to Antiretroviral Therapy among HIV-Positive Individuals in Kenya: Results from the Second Kenya AIDS Indicator Survey, 2012

**DOI:** 10.1371/journal.pone.0167465

**Published:** 2016-12-01

**Authors:** Irene N. Mukui, Lucy Ng’ang’a, John Williamson, Joyce N. Wamicwe, Shobha Vakil, Abraham Katana, Andrea A. Kim

**Affiliations:** 1 National AIDS & STI Control Programme, Department of Preventive and Promotive Health Services, Ministry of Health, Nairobi, Kenya; 2 Division of Global HIV/AIDS, US Centers for Disease Control and Prevention, Nairobi, Kenya; Duke University School of Medicine, UNITED STATES

## Abstract

**Introduction:**

Understanding the levels and associated factors of non-adherence to antiretroviral therapy (ART) is crucial in designing interventions to improve adherence and health outcomes of ART. We assessed non-adherence to ART among HIV-infected persons reporting ART use in a nationally representative survey in Kenya.

**Methods:**

The Kenya AIDS Indicator Survey 2012 was a population-based, household survey of persons aged 18 months-64 years conducted in 2012–2013. Self-reported information was collected on demographics, sexual behaviour, HIV status, and ART use. Blood was collected for HIV testing, and if HIV infected, CD4 and viral load testing. HIV-positive specimens were tested for the presence of antiretroviral (ARV) drugs using a qualitative ARV assay using liquid chromatography-tandem mass spectrometry. HIV-positive persons who reported receiving ART but did not have the ARV biomarker present were defined as being non-adherent to their ARV medication. We restricted our analysis to HIV-infected persons aged 15–64 years who reported receiving ART and had laboratory-confirmed results from ARV testing. Multivariate logistic regression was used to identify variables associated with non-adherence.

**Results:**

A total of 648 (5.6%; CI 4.9–6.3) tested HIV-positive of whom 559 (86.3%) had sufficient volume of blood to be tested for ARV drugs. Of those, 271 (47.7%; CI 41.8–53.6) self-reported HIV-positive status during the interview and 186 (69.1%; CI 62.2–76.0) of those reported taking ART. The ARV biomarker was absent in 18 of 186 individuals (9.4%; CI 4.9–13.8) who thus were defined as being non-adherent to ART. Non-adherence was associated with being aged 15–29 years (AOR 8.39; CI 2.26–31.22, p = 0.002) compared to aged 30–64 years, rural residence (AOR 5.87; CI 1.39–25.61, p = 0.016) compared with urban residence and taking recreational drugs in the past 30 days (AOR 5.89; CI 1.30–26.70, p = 0.022).

**Conclusion:**

Overall, less than 10% of Kenyans aged 15–64 years on ART were not adhering to their HIV medication, highlighting the success of the Kenyan national ART program. Our findings, however, point to the need for targeted interventions particularly for young persons, those in rural areas to improve adherence outcomes, as well as delivery of treatment programs that include psychosocial support as a preventative measure to minimize substance abuse and the risk of treatment failure.

## Introduction

By year-end 2014, approximately 37 million people were living with the human immune-deficiency virus (HIV) globally, with nearly all from low- and middle-income countries. Of those, an estimated 15 million HIV-infected persons were receiving antiretroviral drug (ARV) therapy (ART), a doubling of numbers on ART from 2010 [1]. The use of ART has significantly reduced morbidity and mortality over time in persons living with HIV. Globally in 2014, 1.2 million persons died from AIDS-related causes, representing a 42% reduction since the peak in AIDS deaths in 2004[1].

According to the national HIV estimates for Kenya, 1.4 million adults were living with HIV in 2013 [2]. Of these, approximately 760,694 adults were estimated to be eligible for ART in 2013, of whom 548,588 were receiving ART by year-end 2012[2, 3]. These figures suggest that 72% of adults in need of ART were receiving it, representing more than a 60-fold increase in patients on ART since the introduction of the national HIV treatment program in Kenya in 2003. Increased access to treatment has improved survival and quality of life. In Kenya, 380,000 deaths were estimated to have been averted due to ART between 2000 and 2013[2].

High levels of adherence to ART are needed to ensure optimal benefits of viral suppression and prevention of emergence of HIV drug resistant virus [4]. Adherence to ART has been shown to be a strong predictor of increase in CD4 count after initiation of ART even in persons starting treatment at low CD4 levels [5]. In addition, adherence to ART and hence, the success of treatment, have other public health benefits such as lowering community viral load and reducing sexual, perinatal and injection-related transmission of HIV [6].

Understanding the prevalence of and reasons for non-adherence to ART among HIV-infected persons are important clinical and public health goals in reversing the HIV epidemic worldwide, particularly as countries move to providing ART to all persons living with HIV irrespective of CD4 levels in line with World Health Organization guidelines on ART [7]. A literature review of published studies on correlates of adherence found that only a few determinants were consistently associated with non-adherence, including adverse drug effects, psychological distress, and lack of social support structures, complexity of and inconvenience of the ART regimens. Socio-demographic characteristics, substance abuse, depression, CD4 cell count, and patient–provider relationships were found to be inconsistently associated with non-adherence [8].

In Kenya, published data on population-based levels of and predictors of adherence for persons taking ART are limited. Using data from the second Kenya AIDS Indicator Survey (2012), this paper describes nationally representative data on rates of non-adherence to ART among HIV-infected persons in Kenya aged 15–64 years and associations with select demographic, behavioural, and clinical characteristics.

## Methods

### Study design, sampling and population

The second Kenya AIDS Indicator Survey (KAIS 2012) was a nationally representative population-based, household survey of adults and children aged 18 months to 64 years conducted from October 2012 to February 2013 The detailed methods for KAIS 2012 are described elsewhere [9]. In brief, the survey utilized a stratified two-stage cluster sampling design to select household clusters from which eligible households were selected. Household and individual questionnaires were administered to consenting respondents to collect self-reported demographic, behavioural, and clinical information. Following the interview, blood samples were collected to test for HIV antibodies at a central laboratory. If the sample was found to be HIV-positive, it was tested further for CD4+ T-cell counts and HIV-1 RNA concentration.

This analysis focuses on a subset of KAIS 2012 respondents that were HIV-infected, aged 15–64 years, self-reported that they had ever received ART and had blood specimen available for ARV drug testing. Due to the low number of children aged 18 months-14 years that were HIV-infected and receiving ART (n = 8), children were not included in this analysis.

### Laboratory measures

Dried capillary blood spot (DBS) and venous blood samples were transported to the National HIV Reference Laboratory in Nairobi several times a week. Dried blood spot specimens were tested for HIV using a serial testing algorithm using the Vironostika HIV-1/2 UNIF II Plus O Enzyme Immunoassay as the screening assay (bioMérieux SA, Marcy l'Etoile, France) and confirmed HIV antibody-positive results with the Murex HIV.1.2.O HIV Enzyme Immunoassay (DiaSorin, SpA, Saluggia, Italy). Specimens testing negative by the screening assay were classified as HIV-negative. Specimens testing positive on the screening and confirmatory assays were classified as HIV-positive. Specimens with discordant results were retested using the same algorithm. Twice-discordant results were tested using polymerase chain reaction to determine the final result.

CD4+ T-cell testing was conducted on HIV-positive venous blood using the BD FACs Calibur flow cytometer (Becton Dickinson Vacutainer Systems, Franklin Lakes, New Jersey, U.S.A). HIV-positive dried blood spot specimens were also tested for HIV RNA concentration (Abbott m2000 Real-Time HIV-1 assay, Abbott Park, Illinois, USA) and stored at -70C for future ARV drug testing. Virologic suppression was defined as HIV RNA concentration <1000 copies per mL.

HIV-positive DBS were transported to an external laboratory at the University of Cape Town in South Africa where they were tested for the presence of ARV drugs with a qualitative ARV assay using liquid chromatography-tandem mass spectrometry. The assay, described elsewhere, has been previously applied in national household surveys in South Africa and Kenya [10–11]. The assay tested for the presence of nevirapine, efavirenz, lamivudine, and lopinavir which were included as one or more drugs in the national standardized first-line or second-line ARV regimens at the time of the survey [12]. In 2012, the preferred first-line regimen was tenofovir (TDF) + lamivudine (3TC) + efavirenz (EFV) or nevirapine (NVP) and second-line regimen was zidovudine (AZT) + 3TC + lopinavir/ritonavir (LPV/r) [12]. For pregnant women or patients who were intolerant to TDF, the recommended first-line regimen was AZT + 3TC + EFV/NVP. Integrase strand transfer inhibitors were not available in Kenya in 2012. The ARV assay cut-off was 0. 0.020 μg/ml. Samples with levels observed greater or equal to 0.020 μg/ml was designated as positive for the ARV analyte while levels below 0.020 μg/ml were deemed negative for the analyte. The number of days post ingestion to the ARV assay cut-off was 12–28 days for EFV, 8–9 days for NVP, 1.5 days for 3TC, and 1.5–2.5 days for LPV. Specimens that tested positive for one or more ARV drugs were classified as having the ARV drug biomarker.

### Adherence measures

Non-adherence to antiretroviral (ARV) drugs was the primary outcome in our analysis. To measure non-adherence to ARVs, we analysed data for HIV-infected respondents who self-reported 1) HIV-positive status; 2) that they were receiving ART at the time of the survey; and 3) had specimen available to test for an ARV drug biomarker. Individuals who reported receiving ART but did not have the ARV biomarker present were defined as being non-adherent to ARVs. A self-reported measure of non-adherence based on the question: “In the past 30 days, have you missed taking any of your ARV pills?” was also compared to the ARV biomarker results but not used to define the primary outcome of non-adherence to ARVs in this analysis. Predictor variables assessed included socio-demographic characteristics, including sex, educational level, marital status, wealth, and residence; partner and behavioral characteristics, including HIV status of partner, being sexually active, number of partners in the past year, condom use at last sex in the past year, history of transactional sex and anal sex, symptoms of sexually transmitted infections in the past year, and recent recreational drug use (defined as having taken any illicit oral drugs in the past 30 days); and clinical characteristics, including concomitant receipt of TB treatment, cotrimoxazole, and nutritional supplements (i.e., therapeutic or supplemental feeds and multivitamins), receipt of a HIV care box (i.e., water purifier, plastic clean water vessel, water filter cloth, insecticide treated mosquito net, condoms, and educational materials), frequency of HIV clinic visits andCD4+ T-cell count.

### Data analysis methods

We analysed all data using survey procedures in SAS version 9.3 (SAS Institute, Cary, NC). Estimates presented in these analyses were weighted to account for the sampling design and nonresponse. Frequencies and 95% confidence intervals (CI) were calculated to describe ART adherence status by select characteristics. Statistical significance in cross tabulations was assessed using the Rao-Scott chi-square test. We applied univariate logistic regression to identify potential variables associated with non-adherence to ARVs. Variables selected for univariate logistic regression represented demographic, behavioral, and clinical indicators considered to potentially increase the likelihood of non-adherence or have a higher likelihood of occurring as a result non-adherence to ART. Of note, cotrimoxazole use and anal sex were not included in the univariate model because there were zero observations for non-adherence among individuals who reported anal sex or who did not report receiving cotrimoxazole. In addition, CD4 cell count was not included the model due to the large number of missing observations. Variables with a p-value <0.20 in univariate logistic regression were entered into a multivariate model to identify independent and significant correlates of non-adherence to ART. Variables remaining in the model with a p-value <0.05 were considered statistically significant associations. Odds ratios (OR), adjusted odds ratios (AOR), and 95% confidence intervals (CI) were presented to describe the results of the regression analyses. To extrapolate the estimated number of persons aged 15–64 years on ART who were not adherent to their ARV drugs in the broader population, we applied non-normalized survey weights to the outcome variable and rounded estimates to the nearest 1,000. Non-normalized weights were based on the 2012 projected population data in the 2009 Kenya Population and Housing Census [13].

### Ethical approval

This survey protocol was reviewed and approved by the Institutional Review Boards of the Kenya Medical Research Institute and the U.S. Centres for Disease Control and Prevention, and the Committee on Human Research of the University of California, San Francisco. All participants provided verbal informed consent and had the choice to consent separately to the interview, the blood draw, HIV testing and the storage of their specimens for future testing. Women and men aged 18 to 64 years were eligible to participate in the survey, provided they gave informed consent. For minors aged 10 to 17 years, parental or guardian consent and minor assent were both required for participation in the interview and the blood draw. Parental or guardian consent was required for children aged 18 months to 9 years to participate in the blood draw. For those less than age 18 years who were married, had children, or pregnant, they were considered as emancipated minors, using the criteria from the Kenya 2006 HIV Prevention and Control Act, and were able to provide their own consent to participate in the adult interview and blood draw. The study investigators obtained waiver of documentation of informed consent under 45 CFR 46.117 (c) for all survey participants on the grounds of minimal risk of harm to the subjects. Interviewers documented consent in each study participant’s questionnaire. Verbal informed consent with a signature of the interviewer as documentation of consent was approved by the institutional review boards.

## Results

**Fig 1:**
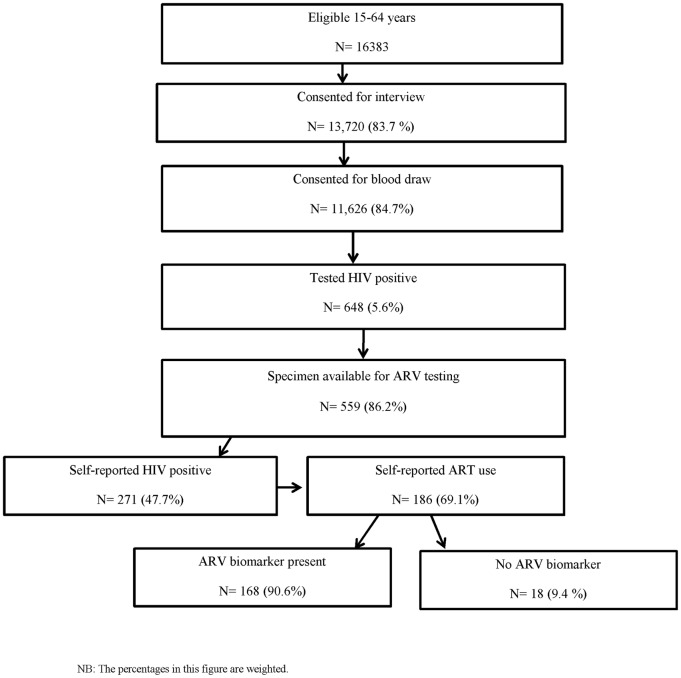
Analysis flow chart among adults and adolescents aged 15–64 years in the Kenya AIDS indicator Survey 2012. The figure presents the flow of how eligible persons for this analysis were derived from the overal Kenya AIDS Indicator Survey. Percentages in this figure are weighted. ART, antiretroviral therapy; ARV, antiretroviral.

A total of 16,383 adult and adolescents aged 15–64 years were eligible for the survey, of whom 13,720 (83.7%) consented to an interview and 11,626 (84.7%) of those consented to the blood draw Fig 1. Among those who provided blood, 648 (5.6%; 95% CI 4.9–6.3) tested HIV-positive in the survey, and of those, 559 (86.3%) had specimens available for ARV testing. Among those, 271 (47.7%; CI 41.8–53.6) self-reported HIV-positive status during the interview and 186 (69.1%; CI 62.2–76.0) of those reported that they were currently receiving ART to treat their HIV infection.

Among those that reported receiving ART, 9.4% (CI 4.9–13.8) did not have any ARV drugs detected and were classified as being non-adherent to their ARV drugs. Extrapolated to the population, this represented 32,000 (CI 16,000–48,000) persons aged 15–64 years on ART who were not adhering to their medication in 2012. In comparison, 14.7% (CI 8.7–20.6) self-reported that they had missed taking any of their ARV pills in the past 30 days, representing 50,000 (CI 29,000–72,000) persons on ART.

The proportion of persons on ART that were non-adherent by select socio-demographic, behavioral, and clinical characteristics are shown in Table 1. Higher levels of non-adherence were observed for younger persons aged 15–29 years (28.7% (CI10.3–37.0) compared with persons aged 30 years and older (6.7%; CI 2.7–10.6). Persons with primary or lower level of education (14.0%; CI 6.2–21.8), in the poorest wealth category (13.7%; CI 5.3–22.1), or residing in rural areas (14.4%; CI 7.4–21.5) had higher levels of non-adherence than persons with higher education (5.7%; CI 0.4–11.0), in the richest wealth category (6.2%; CI 0.6–11.7), or who lived in urban areas (2.3%; CI 0–5.4).

**Table 1:** Non-adherence to ARV drugs among HIV-infected persons receiving ART by select characteristics, Kenya, 2012.

	HIV-infected persons receiving ART	HIV-infected persons receiving ART that were non-adherent to their ARV drugs	
Select characteristics	Unweighted, N	Unweighted, n	Weighted % (95% CI)^a^
**Total**	186	18	9.4 (4.9–13.8)
**Demographic characteristics**			
**Sex**			
Male	45	4	10.4 (0.5–20.3)
Female	141	14	8.9 (4.2–13.5)
**Age category (years)**			
15–29	25	7	28.7 (10.3–47.0)
30+	161	11	6.7 (2.7–10.6)
**Educational level**			
Complete primary or below	86	12	14.0 (6.2–21.8)
Secondary+	100	6	5.7 (0.4–11.0)
**Marital status**			
Not currently married	99	10	9.2 (3.2–15.2)
Currently married	87	8	9.5 (2.8–16.3)
**Wealth index**			
Poorest	77	13	13.7 (5.3–22.1)
Middle	39	1	5.5 (0.0–15.0)
Richest	70	4	6.2 (0.6–11.7)
**Residence**			
Rural	105	16	14.4 (7.4–21.5)
Urban	81	2	2.3 (0.0–5.4)
**Sexual partners and risk behavior**			
**HIV-positive partner in past year**			
No	145	16	11.4 (5.6–17.2)
Yes	41	2	3.8 (0.0–9.1)
**Sexually active in past year**			
No	65	8	10.8 (3.0–18.5)
Yes	121	10	8.7 (3.0–14.4)
**Multiple partners in past year**			
No	169	17	9.3 (4.7–14.0)
Yes	17	1	9.4 (0.0–27.2)*
**Condom use at last sex in past year**			
No	124	14	10.4 (4.8–16.0)
Yes	62	4	7.4 (0.4–14.4)
**Ever received money, gifts, or favors in exchange for sex**			
No	168	17	9.3 (4.7–13.8)
Yes	18	1	10.0 (0.0–29.1)*
**Ever gave money, gifts, or favors in exchange for sex**			
No	167	15	7.7 (3.8–11.5)
Yes	19	3	21.0 (1.5–40.5)*
**Ever engaged in anal sex**			
No	180	18	9.7 (5.1–14.3)
Yes	6	0	-
**Recreational drug use in past year**^b^			
No	168	15	7.2 (3.5–10.9)
Yes	18	3	28.8 (2.2–55.4)*
**Clinical characteristics**			
**Symptoms of sexually transmitted infection in past year**			
No	159	14	8.4 (3.9–12.9)
Yes	27	4	15.2 (0.4–30.1)
**Currently on TB treatment**			
No	180	16	8.7 (4.4–12.9)
Yes	6	2	28.7 (0.0–71.6)*
**Currently taking cotrimoxazole**			
No	3	0	-
Yes	182	18	9.6 (5.0–14.1)
**Currently taking nutritional supplements**			
No	135	15	10.5 (5.0–16.1)
Yes	51	3	6.3 (0.0–13.3)
**Received a HIV care box**			
No	105	10	8.1 (2.7–13.5)
Yes	81	8	10.8 (3.8–17.8)
**Last HIV clinic attendance**^c^			
Within the last 1 month	131	12	8.3 (3.3–13.4)
Within the last 2 or more months	48	5	11.5 (0.5–22.4)
**CD4 count <350 cells/mm3**^c^			
No	73	5	5.1 (0.0–10.6)
Yes	31	4	10.2 (0.7–19.7)
**Suppressed viral load HIV RNA concentration <1000 copies/mL)**			
No	45	15	32.0 (16.4–47.6)
Yes	140	3	2.3 (0.0–5.0)

^a^Estimates were weighted to into account for sampling probability and participant nonresponse.

*Estimate may be unreliable due to small denominator (<25 observations).

^b^Recreational drug use was defined as taking any illicit oral or injected drugs in the past 12 months.

^c^There was 1 missing observation for the variable “Last HIV care clinic attendance” and 9 missing observations for the variable “CD4 count <350 cells/mm^3^”.

When considering sexual partners, levels of non-adherence to ARVs was three times higher among persons who did not have an HIV-positive partner in the past year (11.4%; CI 5.6–17.2) compared with those who had a HIV-positive partner in the past year (3.8%; CI 0–9.1). Additionally, persons engaging in high-risk behavior, including a history of giving money, gifts, or favors in exchange for sex (21%; CI 1.5–40.5) and using recreational drugs in the past month (28.8%; CI 2.2–55.4) had higher levels of non-adherence to ARV drugs than persons who had never given money, gifts, or favors in exchange for sex (7.7%; CI 3.8–11.5) and persons who had not used recreational drugs in the past month (7.2%; CI 3.5–10.9). Persons who reported being concurrently on medication to treat tuberculosis (TB) disease had non-adherence levels of 28.7% (CI 0–71.6) compared with 8.7% (CI 4.4–12.9) of persons who were not taking TB medication. A higher proportion of non-adherent persons had viral load above 1000 copies/ml, (32%; CI 16.4–47.6) compared to those who were adherent (2.3%; CI 0–5.0).

In univariate logistic regression, the variables that met the criteria for inclusion in multivariate logistic regression were age (p = 0.001), education (p = 0.136), residence (p = 0.011), HIV status of partner (p = 0.135), a history of giving money, gifts, or favors in exchange for sex (p = 0.075), using recreational drugs in the past year (p = 0.021), having anal sex (p = 0.001) and concurrent TB treatment (p = 0.182) (Table 2). After controlling for these variables in multivariate analysis, being aged 15–29 years (AOR 8.39; CI 2.26–31.22) compared to aged 30–64 years, rural residences (AOR 7.21; CI 1.47–35.26) compared to urban residences, and using recreational drugs in the past year (AOR 5.20; CI 1.29–20.98) were significantly and independently associated with higher adjusted odds of non-adherence to ARV drugs. We found no significant associations between non-adherence and education, marital status, wealth, current sexual activity, HIV status of partner, number of partners, condom use, a history of receiving money, gifts, or favors in exchange for sex, a history of anal sex, concomitant receipt of TB treatment, nutritional supplements, and HIV care box and last HIV clinic visit.

**Table 2:** Factors associated with non-adherence to ARV drugs among HIV-infected persons receiving antiretroviral therapy, Kenya, 2012.

	Unadjusted OR (95%CI)	p-value	Adjusted OR (95% CI)	p-value
**Select characteristics**				
**Demographic characteristics**				
**Sex**				
Men	1.19(0.36–3.98)	0.774		
Women	Ref			
**Age category (years)**				
15–29	5.64(1.98–16.04)	0.001	8.39 (2.26–31.22)	0.002
30+	Ref		ref	
**Educational level**				
Complete primary or lower	2.68(0.81–8.90)	0.107	3.67 (0.96–14.01)	0.058
Secondary+	Ref		ref	
**Wealth index**				
Poorest	2.41(0.68–8.50)	0.296		
Middle	0.88(0.11–7.14)			
Richest	Ref			
**Residence**				
Rural	7.25(1.57–33.40)	0.011	5.98 (1.39–25.61)	0.016
Urban	Ref		ref	
**Marital status**				
Not currently married	Ref	0.944		
Currently married	1.04(0.36–3.02)			
**Sexual partners and behavior**				
**HIV-positive partner in past year**				
No	3.25(0.69–15.28)	0.135	5.61 (0.46–68.41)	0.177
Yes	Ref		ref	
**Sexually active in past year**				
No	Ref	0.677		
Yes	0.79(0.26–2.39)			
**Multiple partners in past year**				
No	Ref	0.993		
Yes	1.01(0.12–8.80)			
**Condom use at last sex in past year**				
No	Ref	0.537		
Yes	0.69(0.21–2.23)			
**Ever received money, gifts, or favors in exchange for sex**				
No	Ref	0.938		
Yes	1.09(0.12–9.60)			
**Ever gave money, gifts, or favors in exchange for sex**				
No	Ref	0.075	ref	0.642
Yes	3.20(0.89–11.54)		0.72 (0.18–2.87)	
**Ever engaged in anal sex**				
No	Ref	<0.001		
Yes	0.00(0.00–0.00)			
Recreational drug use in past year*				
No	Ref	0.021	ref	0.022
Yes	5.20(1.29–20.98)		5.89 (1.30–26.70)	
**Clinical characteristics**				
Symptoms of sexually transmitted infection in past year				
No	Ref	0.31		
Yes	1.97(0.53–7.26)			
**Currently on TB treatment**				
No	Ref	0.182	ref	0.086
Yes	4.25(0.51–35.59)		3.41 (0.84–13.87)	
**Currently taking nutritional supplements**				
No	1.76(0.47–6.61)	0.401		
Yes	Ref			
**Received a HIV care box**				
No	Ref	0.545		
Yes	1.37(0.50–3.76)			
**Last HIV clinic attendance**				
Within the last 1 month	Ref	0.601		
Within the last 2 or more months	1.43(0.38–5.44)			

*Recreational drug use was defined as taking any illicit oral or injected drugs in the past 12 months.

## Discussion

Using an objective measure of non-adherence to ART, this nationally representative population-based survey confirmed that less than 10% of HIV-infected Kenyans aged 15–64 years receiving ART or an estimated 32, 000 persons were not appropriately adhering to their HIV treatment. This low level of non-adherence to ART highlights the success of the national ART program in Kenya, where non-adherence levels are lower than reported in other sub-Saharan African countries, including: Rwanda, where 23% of ART patients were non-adherent based on 30-day recall [14]; Ghana, where 14% of ART patients were non-adherent [15]; rural Zambia where 40% of ART patients were not adherent [16], and a pooled analysis of African adherence studies, where overall non-adherence levels were reported to be 23% [17]. Data from developed countries also suggest higher non-adherence levels, with estimates of 45% in a pooled analysis of adherence studies in North America [17], 37% in Sweden [18], 34% in Brazil [19], and 43% in Spain [20]. Studies that have combined indicators of dose, timing, food and other measures have also generally reported higher non-adherence rates, [21–22]. The vast majority of these external studies, however, were not nationally-representative and are based on self-reports, limiting the extent to which the findings can be compared with those in our study.

Since 2005, Kenya has implemented numerous interventions to provide treatment support and enhance ARV drug adherence among patients on ART, which have likely contributed to the high level of adherence we observed among the population of people receiving ART. These interventions include use of community and peer support systems, clinic appointment systems to enhance adherence as well as innovative use of short message service (SMS) via mobile devices [23, 24].

Moreover, the detection of ARV biomarkers in the blood of individuals testing HIV-positive and self-reporting ARV use in our study was lower than the reported levels of self–reported non-adherence using a 30-day recall indicator. Had our results relied on self-report for defining non-adherence, population estimates of non-adherence would be overestimated by approximately 20,000 persons. Although there are limited data comparing self-reported adherence with plasma levels of ARV drugs, a study comparing the measured plasma levels of protease inhibitors with patients’ self-reported adherence found that patients who had concentrations below the assay limit of quantitation correlated with patient self-reported non-adherence in the previous day [25]. A second study that assessed adherence to ART among a clinical cohort found deviation from the taking ART regimens as per time and dietary schedule was associated with decreased plasma drug levels, and a decreased likelihood of having viral suppression [26]. Nonetheless, self-reported adherence is susceptible to recall and social desirability bias and has been documented to produce estimates of adherence that are up to 10–20% higher than those from electronic drug monitoring [27, 28]. However, our study indicates the converse where self-reported adherence over-estimates non-adherence compared to measurement of non-ART adherence based on absence of the ARV biomarker.

We found correlates of non-adherence to ART, providing important epidemiologic insight on sub-populations where the national ART programme should target to improve long-term adherence and clinical outcomes on treatment. In our analysis, adolescents and young adults were more likely to be non-adherent to ART. Our findings are supported by those from a large observational study comparing adherence among adolescents and adults across 9 sub-Saharan African countries. This study found adolescents 50% times less likely to adhere to ART compared to adults [29]. High levels of non-adherence among adolescents have also been reported in developed country settings [30]. Adolescents and youth face numerous social, economic, cultural, individual level and treatment related challenges that may contribute to the higher rates of non-adherence observed [31,32]. These include but are not limited to stigma, challenges with disclosure, mental health problems, poverty and medication-related barriers such as pill burden and side effects [30–32]. Our study also found significant correlation between non-adherence and rural residence. Previous studies in Kenya suggest there may be differences in adherence levels across urban/rural settings where non-adherence among patients on treatment has been reported as low as 18% in an urban slum in Nairobi [33] and as high as 23% in rural clinics in western Kenya [34]. A Zambian study assessing social factors associated with adherence found that rural-related conditions, including limited options for remembering to take and when to take ARV medication, may affect adherence [35]. Additionally, the association between non-adherence and rural areas may indicate difficulties in access to health services [36]. Our findings support the need for regional evaluation to assess factors and different components of the treatment program including access, adherence counselling and support for patients in rural settings.

We also found that ART patients who reported recreational drug use in the past year were significantly more likely to be non-adherent to their ARV drugs compared with those who did not report this drug use. Substance abuse can interfere with the ability of persons to take their ART medication as instructed. This finding is supported by other studies that have reported that the concomitant use of one or more illicit drugs with ART can negatively affect adherence to treatment [8, 37–39]. With the increases in illicit drug use in Kenya [40], there is need to address substance abuse through expansion of programs for identification and management of recreational drug use to minimize the negative effects on adherence and ultimately viral suppression.

This analysis should be considered in light of its limitations. Our estimates of non-adherence to ART relied on self-reported HIV-positive status, and if reported HIV-positive, receipt of HIV care and treatment services. Under-reporting of HIV status due to concerns around disclosure would have led to under-reporting of received HIV care and treatment services, affecting our estimates of non-adherence on ART. Because we did not collect information on length of time on ART or type of ARV medication taken, we were not able to assess whether non-adherence was affected by time on ART or specific ARV medications. Additionally, over 50% of HIV-positive venous samples were hemolyzed during transport to the centralized laboratory. However, persons without CD4+ T-cell count results were similar in age, sex, regional distribution compared with those with CD4+ T-cell count results. We therefore anticipate minimal bias in our estimates that report on CD4+ T-cell count data. The number of persons who self-reported ART use and classified as non-adherent to ARV drugs based on results from the ARV biomarker was small. Due to the small sample size, only major correlates were likely to be significantly associated with non-adherence to ART, while other potential correlates may not have been significant due to the low power to detect differences. Confidence intervals around estimates and associations are also wide; therefore, results should be interpreted cautiously. Finally, because KAIS 2012 was a cross-sectional study, it was not possible to confirm temporality and causality of associations observed.

Despite these limitations, we present nationally representative data on the level of non-adherence to ART in Kenya which have not been presented elsewhere. Using an objective measure of non-adherence to medication, these data indicate that the levels of non-adherence were low, demonstrating substantial achievement of Kenya’s large HIV treatment program which had over 500,000 adults receiving ART in 2012 [3]. To minimize the risk of treatment failure among ART patients, the Kenyan HIV treatment program should continue to routinely monitor the levels of adherence to ART and factors associated with sub-optimal adherence at both a national and sub-national level. The results of this surveillance should be used to tailor strategies for the different subpopulations with suboptimal adherence. Specifically, determining and addressing barriers to adherence among rural populations, adolescents and youth and persons using illicit drugs is critical in achieving the full benefits of antiretroviral therapy, nationally and among different sub-populations. Simplified approaches for monitoring ARV adherence among ART patients with particular benefit to rural settings and among young persons, including periodic measurement of ARV drug concentration in hair, may also be considered [41,42].
